# A Purification Method for a Molecular Complex in Which a Scaffold Molecule Is Fully Loaded with Heterogeneous Molecules

**DOI:** 10.1371/journal.pone.0120576

**Published:** 2015-03-17

**Authors:** Shoji J. Ohuchi, Fumihiko Sagawa, Hirohisa Ohno, Tan Inoue

**Affiliations:** Laboratory of Gene Biodynamics, Graduate School of Biostudies, Kyoto University, Kyoto, Japan; National Taiwan University, TAIWAN

## Abstract

An affinity resin-based pull-down method is convenient for the purification of biochemical materials. However, its use is difficult for the isolation of a molecular complex fully loaded with multiple components from a reaction mixture containing the starting materials and intermediate products. To overcome this problem, we have developed a new purification procedure that depends on sequential elimination of the residues. In practice, two affinity resins were used for purifying a triangular-shaped RNP (RNA-protein complex) consisting of three ribosomal proteins (L7Ae) bound to an RNA scaffold. First, a resin with immobilized L7Ae protein captured the incomplete RNP complexes and the free RNA scaffold. Next, another resin with an immobilized chemically modified RNA of a derivative of Box C/D motif, the binding partner of L7Ae, was used to capture free protein. The complete triangular RNP was successfully purified from the mixture by these two steps. Obviously, the purified triangular RNP displaying three protein-binding peptides exhibited an improved performance when compared with the unrefined product. Conceptually, this purification procedure should be applicable for the purification of a variety of complexes consisting of multiple components other than RNP.

## Introduction

Currently, molecular complexes consisting of various, multiple biomaterials have been developed [1–6]. These complexes are new bioengineering tools for manipulating cellular functions in a highly sophisticated manner. They take advantage of multi-functional materials, such as proteins attached to another molecule that serves as a scaffold. In their most effective configurations, the scaffold must be fully loaded with the desired functional molecules. However, it is often inevitable that intermediate complexes containing fewer functional materials are produced, which results in reduced performance when compared with a fully loaded scaffold. As yet, a simple and efficient purification method for the fully loaded complex has not been described. In this paper, we describe a convenient and generally applicable purification method that employs two affinity resin-based systems applied consecutively. As an example, we purified an RNA-protein complex to demonstrate the utility of this method.

Sophisticated artificial RNA-protein complexes with defined three-dimensional (3D) structures have been designed and constructed by combining the two substances. Previously, an RNP nano-object consisting of three L7Ae proteins bound to an RNA scaffold was constructed by employing L7Ae, a ribosomal protein bound to an RNA molecule containing a box C/D kink-turn motif [5]. Binding of the protein causes the box C/D motif in the RNA at three sites to bend by 60° to form a nearly equilateral triangle. The triangular RNP becomes functional if the L7Ae protein is fused to another functional protein [5, 6]. For example, the regulation of receptor oligomerization was achieved by controlling the size of the RNP containing the corresponding three ligands at the tips: the distance between the ligands is determined by the size of the triangular-shaped scaffold RNA [6].

The triangular RNP can be prepared by mixing the RNA and the protein components containing L7Ae, resulting in an unrefined RNP. However, the purification of the unrefined product is cumbersome because the reaction mixture contains the fully loaded triangular RNP together with partially formed RNP complexes carrying one or two L7Ae proteins, as well as the starting materials. It is conceivable that this contamination might affect the performance of the triangular RNP adversely, so that it is difficult to determine the actual performance of the fully loaded RNP.

In this study, we demonstrate a procedure for the purification of the fully loaded triangular RNP by eliminating the incomplete complexes and the starting materials by the successive use of affinity resins. Polyacrylamide gel electrophoresis based separation followed by RNP elution should be adequate for the purification [5, 6]. Alternatively, high-performance liquid chromatography might also be applicable. However, our procedure is more convenient and less time consuming. Furthermore, our method might be applicable for the purification of other complexes consisting of multiple molecules as well.

## Materials and Methods

### Protein preparation

L7Ae and L7Ae fusion proteins were expressed and purified as previously described [5, 6]. For the expression of the L7Ae-Strep-tag II (ST2)-fusion protein [7], a synthetic DNA fragment coding ST2 sequence [5’-gaattcaagg tggcAGCAAC TGGTCCCATC CGCAGTTTGA GAAGggtggc ctcgag-3’ (*Eco*RI and *Xho*l restriction sites and the ST2 coding region are indicated by underline and uppercase letters, respectively)] was inserted into *Eco*RI and *Xho*l sites in an L7Ae expression plasmid, pET28b-His-L7Ae-cmyc-His. All synthetic DNAs were purchased from Operon Biotechnologies (Japan).

### RNA preparation

The RNA components of the triangular RNP were prepared as previously described [5, 6]. For the preparation of 35-nucleotide (nt) fragments of the box C/D RNA and its derivatives, partially double-stranded synthetic DNAs were used as templates for *in vitro* transcription [8]. The transcription of RNAs containing 2’-fluoro-modified pyrimidine (2’-fPy-RNA) was carried out using a DuraScribe T7 transcription kit (Epicentre).

### Surface plasmon resonance (SPR) analysis

SPR analysis was carried out as described previously [9].

### Affinity resin preparation

The affinity resins were prepared by the canonical amine-coupling reaction using NHS-activated Sepharose 4 Fast Flow (GE Healthcare) according to the supplier’s protocol. Typically, 750 pmol L7Ae was immobilized onto 1 μL of the resin. Chemically synthesized BCD35g 2’-fPy-RNA (fBCD35g) with a 5’-amino modification was purchased from GeneDesign (Japan), and typically, 80 pmol of the RNA was immobilized onto 1 μL of the resin. The prepared L7Ae- and fBCD35g-immobilized resins were stored in 20% ethanol and in the assay buffer [20 mM HEPES-KOH (pH 7.5), 150 mM KCl, 1.5 mM MgCl_2_, 2 mM DTT, and 3% glycerol], respectively, at 4°C until use.

### Fluorescence labelling of L7Ae

Labeling of L7Ae was carried out by the amine coupling of the fluorescence moiety to the L7Ae protein. For the coupling reaction, 400 μM L7Ae was incubated with 2 mg/mL Alexa Fluor 633 Succinimidyl Ester (Invitrogen) in the assay buffer at room temperature for ~100 min. The reaction was stopped by adding an equal volume of 1 M Tris-HCl (pH 7.6), followed by incubation at room temperature for 1 hour. The uncoupled fluorescence moiety was removed by ultrafiltration using Amicon Ultra 0.5-mL centrifugal filters-10K (Merck Millipore). Because the labeling reaction generated a non-negligible percentage of the inactive protein, the active population of the labeled L7Ae molecules was selectively recovered by using fBCD35g-immobilized resin. The labeled protein sample containing 10 nmol of the labeled L7Ae was applied to 2.5 μL of the resin and incubated in the assay buffer with gentle shaking at room temperature for 30 min. Then, the resin was washed with the assay buffer, followed by elution of the bound protein with 100 mM NaOH. The eluate was immediately neutralized by adding an equal volume of 100 mM HCl, followed by a buffer exchange via ultrafiltration as described above. To estimate the concentration of the labeled protein, a serial dilution of the protein sample was subjected to an electrophoretic mobility shift assay (EMSA) with Tri26L/S RNA, as reported previously [5, 6]. A known concentration of unlabeled L7Ae was subjected to EMSA as a concentration standard.

### Purification of the triangular RNP

Using the fluorescently labelled L7Ae and Tri26L/S RNA, the triangular RNP samples were prepared as previously described [5, 6]. Prior to use, the affinity resins were washed 3 times with water and the assay buffer. Initially, 10–30 μL of the samples were applied to 3-μL of L7Ae-immobilized resin and incubated with gentle shaking at room temperature for 15 min. Then, the supernatant was recovered by a quick spin, followed by application to 3-μL of fBCD35-immobilized resin. After 5 min incubation with gentle shaking at room temperature, the supernatant was recovered by a quick spin and subjected to analysis. EMSA and atomic force microscopy (AFM) were carried out as previously described [5, 6]. The fluorescent-labelled L7Ae was detected by FLA-7000 (FujiFilm).

### Strep-tag II (ST2)-binding assay

Internally labelled RNAs were prepared by *in vitro* transcription in the presence of [alpha-^32^P]-CTP (PerkinElmer). Using the labelled RNA, 30-μL of a 10-fold concentrate of triangular RNP with L7Ae or L7Ae-ST2 fusion protein was prepared. Then, the RNP samples were subjected to purification as described above. The RNA concentration of the recovered samples was measured by liquid scintillation counting (LS-6500, Beckman Coulter), followed by dilution with the assay buffer to a final RNA concentration of 1 nM. 100-μL of the purified and diluted sample was transferred into Strep-Tactin coated microtiter plates (IBA GmbH, Germany). After 30 min incubation at room temperature, the microtiter plates were washed with 100-μL of the assay buffer twice, followed by RNP elution with 100-μL of 4.5 mM D-desthiobiotin (IBA GmbH) at room temperature for 30 min. The released RNP was recovered, and the remaining bound RNP was recovered by an additional 30-min elution under the same conditions. The first and second eluates were combined, and the total amount of RNA eluted was measured by liquid scintillation counting. EMSA was carried out as described above. For the detection of [^32^P]-labelled RNA, the gels were dried and autoradiographed using a phosphoimager screen. The gel images were scanned by FLA-3000 (Fujifilm).

## Results and Discussion

### Purification method: Sequential elimination of unwanted materials and prematurely formed products

**Fig 1:**
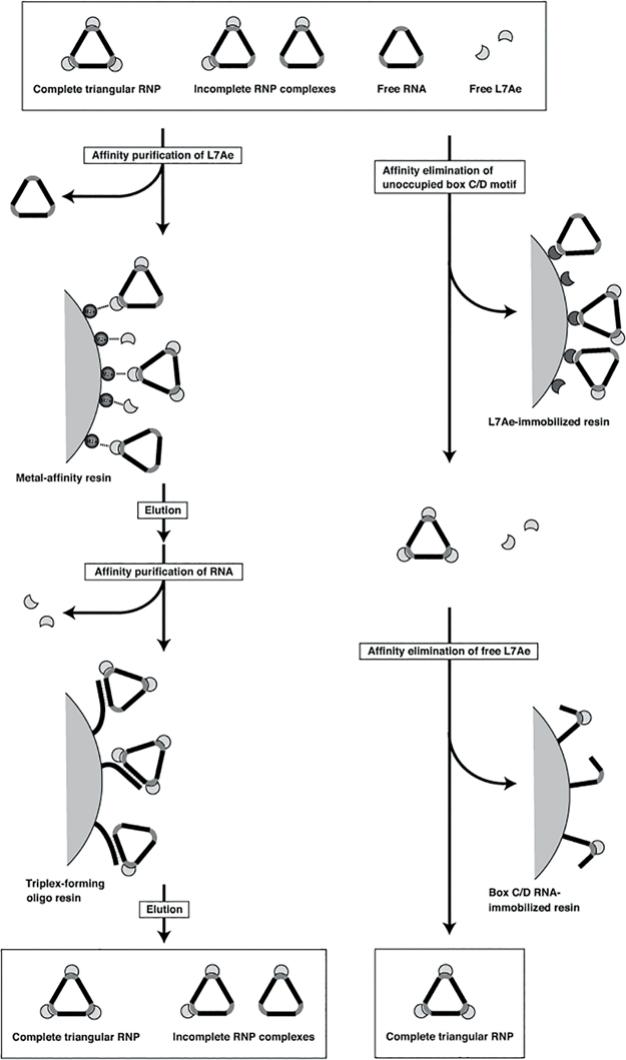
Schematic diagram of the purification procedures for the triangular RNP. Left and Right lines indicate the sequential affinity purification and the sequential affinity elimination procedures, respectively. The sequential affinity purification of the triangular RNP is performed with a metal (*i*.*e*., Ni^2+^ or Co^2+^)-chelated resin to capture the hexa-histidine-tagged L7Ae, followed by treatment with a triplex-forming oligonucleotide-immobilized resin to capture the RNA (left line). The sequential affinity elimination is performed with the L7Ae-immobilized resin to eliminate the incomplete RNP complexes and the free RNA component, followed by elimination of the free L7Ae protein with the box C/D RNA-immobilized resin (right line). Gray and black regions on the RNA indicate the box C/D motif and the stem, respectively.

The affinity resin-based pull-down method is a simple, effective and fast purification procedure for biochemical studies. For the purification of multi-subunit complexes, a sequential affinity purification using affinity resins specific for each component of the mixture is effective for eliminating unwanted starting materials [10]. The purification of the triangular RNP can be performed with a metal (*i*.*e*., Ni^2+^ or Co^2+^)-chelated resin to capture the hexa-histidine-tagged L7Ae, followed by treatment with a triplex-forming oligonucleotide-immobilized resin to capture the RNA (Fig. 1, left line). However, the incomplete RNP complexes carrying one or two L7Ae proteins co-purify with the triangular RNP (Fig. 1, left line).

To solve this problem, we adopted an affinity resin-based method for eliminating the incomplete complexes and the starting materials (Fig. 1, right line). In practice, two affinity resins, one specific for the unoccupied box C/D motif on the RNA and one specific for the free L7Ae, are employed. In the first step, the immobilized L7Ae on the resin captures the incomplete RNP complexes and the free RNA component. In the second step, the free L7Ae protein is excluded by using the box C/D RNA-immobilized resin. Finally, the method enables the purification of the fully equipped triangular RNP (Fig. 1, right line).

### Design of a stable box C/D RNA containing 2’-fluoro-modified pyrimidine

**Fig 2:**
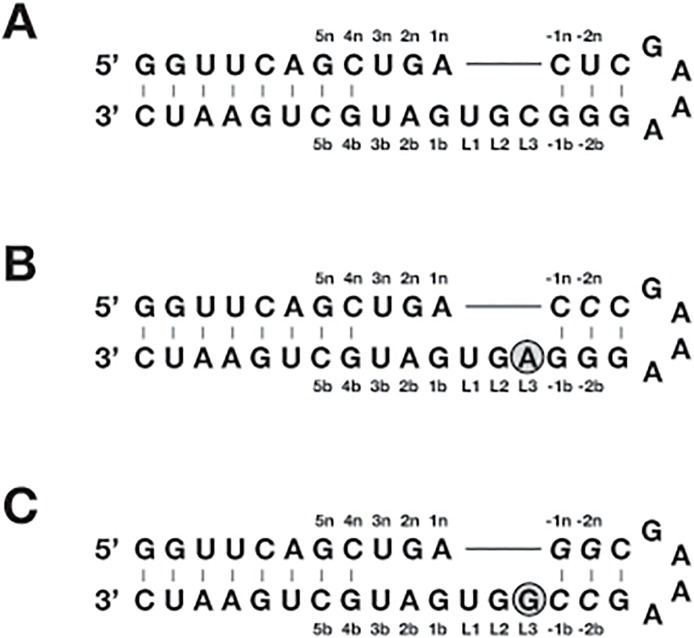
RNAs with original (A) and modified (B and C) box C/D motifs. (A) BCD35 RNA with the original box C/D motif. (B) BCD35a RNA with the C to A base replacement at the L3 position. (C) BCD35g RNA with the C to G base replacement at the L3 position. The replaced bases are indicated by gray circles. Additional base replacements for the retention of two-dimensional structures are indicated by Italics.

L7Ae is a robust protein so the L7Ae-immobilized resin is suitable for long-term storage. However, RNA is chemically unstable, so the box C/D RNA-immobilized resin is unsuitable for long-term storage. To eliminate this problem, we investigated whether 2’-fluoro-modified pyrimidine (2’-fPy), a very popular modification reagent for improving the stability of RNA [11], is applicable for the box C/D motif by employing a 35-nt RNA fragment containing the box C/D motif (BCD35 is abbreviation for the 35-nt RNA containing the box C/D motif) (Fig. 2A). The dissociation constant (K_D_) between BCD35 RNA and L7Ae was determined as 0.29 nM using the surface plasmon resonance (SPR) method employing a L7Ae-immobilized sensor chip and the RNA as the analyte (Table 1, S1 Fig.): The affinity of the RNA was moderately higher than the previously reported value for a shorter RNA molecule with a box C/D motif, most likely due to the elongated stem that stabilizes the secondary structure [9]. When the 2’-fPy-RNA modified BCD35 was analyzed by SPR, no binding signal was observed even when a high concentration (up to 2 μM) of the RNA was injected, indicating that the 2’-fPy modification severely reduced the affinity to L7Ae (Table 1, S1 Fig.).

**Table 1:** Kinetic parameters of the original and modified box C/D motifs.

RNA	ka [/M/sec]	kd [/sec]	K_D_ [nM]
BCD35 RNA	2.03 (±0.27) x10^5^	5.41 (±0.93) x10^-5^	0.29 (±0.11)
BCD35a RNA	2.82 (±0.28) x10^5^	3.56 (±0.28) x10^-5^	0.13 (±0.03)
BCD35g RNA	4.14 (±0.43) x10^5^	6.52 (±0.03) x10^-5^	0.17 (±0.07)
BCD35 2’-fPy-RNA	n.d.	n.d.	n.d.
BCD35a 2’-fPy-RNA	2.87 (±0.08) x10^4^	6.88 (±0.42) x10^-4^	22.8 (±0.7)
BCD35g 2’-fPy-RNA	5.26 (±0.26) x10^5^	1.55 (±0.00) x10^-3^	2.96 (±0.14)

Kinetic parameters of the original and modified box C/D motifs. The association rate (ka) and dissociation rate (kd) of the indicated RNA for L7Ae protein were evaluated by SPR analysis with an L7Ae-immobilized sensor chip. The dissociation constant (K_D_) was calculated from the ka and kd values. At least, three independent experiments were carried out, and the average values are tabulated. “n.d.” means not detectable.

**Fig 3:**
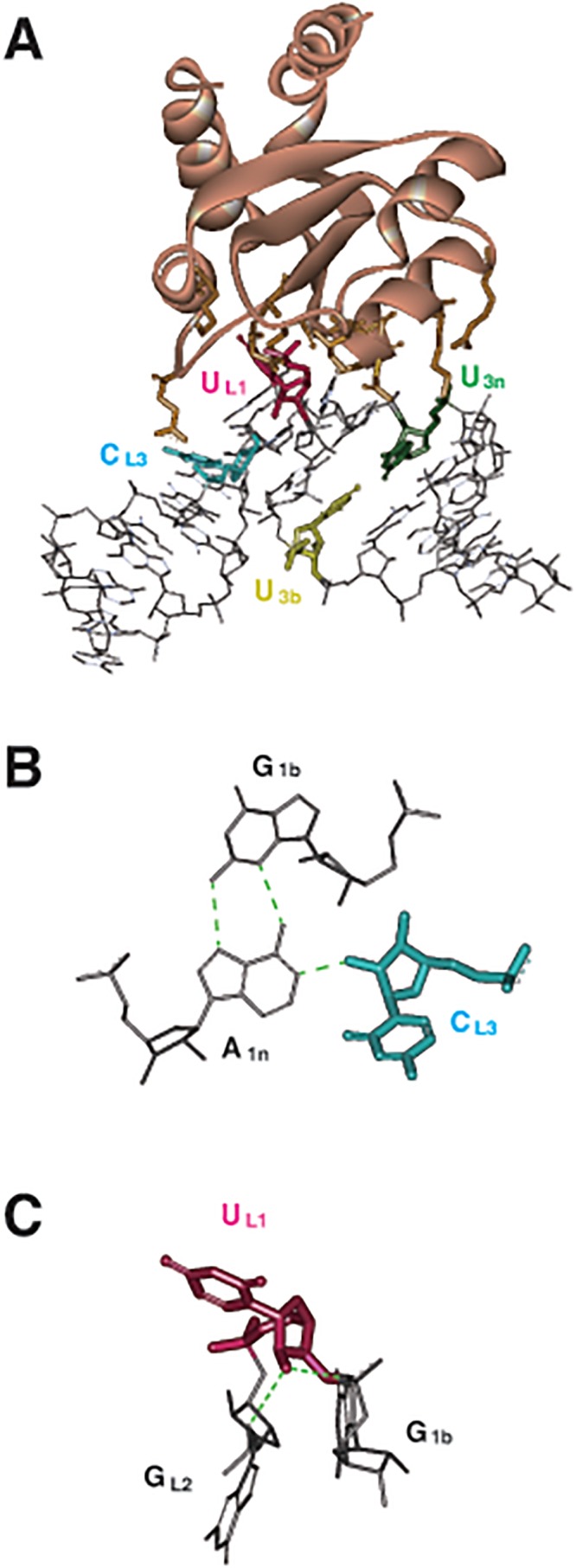
3D structure of the complex of L7Ae protein and the box C/D RNA. (A) Crystal structure of the L7Ae-box C/D complex (PDB ID 1RLG). L7Ae protein and the box C/D RNA are shown as ribbon model colored with brown and stick model, respectively. Four pyrimidine residues on the box C/D RNA essential for the interaction with L7Ae protein are indicated by colors. (B) Intramolecular hydrogen-bonds of box C/D RNA including 2’ hydroxyl group of C_L3_. (C) Intramolecular hydrogen-bonds of box C/D RNA including 2’ hydroxyl group of U_L1_. Images were prepared with Discovery Studio (Accelrys).

No 2’ hydroxyl groups of the pyrimidine residues interact directly with the L7Ae protein according to the crystal structure of the L7Ae-box C/D RNA complex [12]. However, the 2’ hydroxyl group of the U residue at the L1 position (U_L1_) and the C residue at the L3 position (C_L3_) form intramolecular hydrogen bonds, suggesting that they have a crucial role in binding (Fig. 3). Replacement of the base C_L3_ with an A or G residue was attempted because the base replacement of U_L1_ severely reduces the affinity due to a direct interaction between the base moiety and L7Ae protein [13]. As expected, the two replacements conferred no negative effects on the affinity (Table 1, S1 Fig.).

For 2’-fPy-RNA, a C to A replacement (BCD35a) showed an affinity two orders of magnitude lower than that of the original BCD35 RNA. However, a C to G replacement (BCD35g) showed an affinity of one order of magnitude lower than the original (Table 1, S1 Fig.). The reduction in affinity was likely caused by the elimination of the intramolecular hydrogen bond including the 2’ hydroxyl group of U_L1_ (Fig. 3C). Alternatively, alteration of physicochemical properties of A-form helixes by the 2’-fPy modifications might affect the affinity [14]: The two synthetic derivatives have different base sequences at the stem, indicating that the hypothesis explains the difference between the two 2’-fPy-RNAs. At least, the K_D_ value of BCD35g 2’-fPy-RNA (fBCD35g) was low enough (3 nM) for the purification of the triangular RNP to be successful.

### Purification of the triangular RNP by a sequential affinity elimination method

Purification of the fully equipped triangular RNP by a sequential affinity elimination method was investigated by employing L7Ae- and fBCD35g-immobilized resins. 0.3 pmol of box C/D RNA was captured with 1 pmol of the immobilized L7Ae, while 1 pmol of L7Ae was captured with 1 pmol of the immobilized fBCD35g (data not shown). The resins were stable under the storage conditions for several weeks (data not shown).

**Fig 4:**
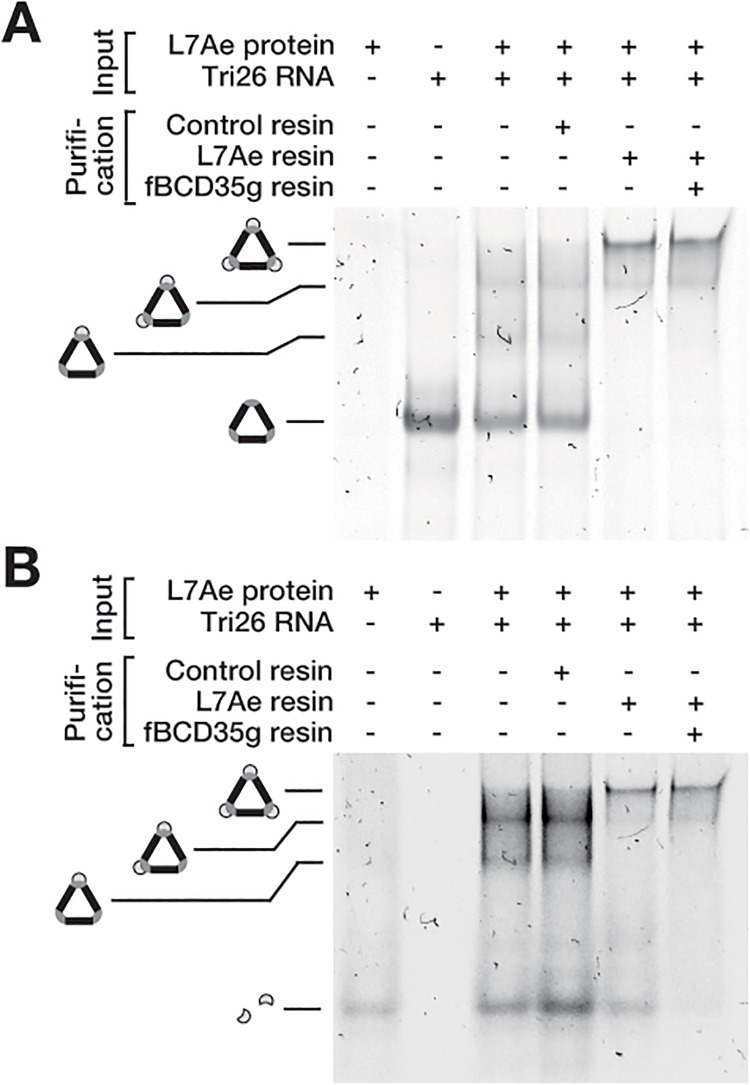
Purification of the triangular RNP by sequential affinity elimination. (A) Analysis of the RNA and RNP complexes by EMSA. The crude RNP samples (3rd lane) were prepared by mixing 150 nM of the Alexa633 labelled L7Ae (1st lane) and 100 nM of Tri26L/S RNA (2nd lane). The RNP samples were subjected to the purification treatment noted above the gel image. The gel was stained with SYBR green to scan for the RNAs. The three upshifted bands in the 3rd and 4th lanes correspond to the RNPs with one, two and three L7Ae molecules bound, respectively. (B) Analysis of L7Ae protein and the RNP complexes by EMSA. The purification treatment was conducted as noted above except that the gel was scanned for the Alexa633 fluorescence.

The triangular RNP was prepared with 100 nM of the RNA component, Tri26L/S, and 150 nM of the fluorescently labelled L7Ae. The complete triangular RNA (4% of the total RNA), incomplete RNP complexes (36% of the total RNA), and free materials were present in the mixture under those conditions (Fig. 4A and 4B, lane 3). First, the sample was applied to the L7Ae-immobilized resin and incubated at room temperature for 15 min. As expected, most of the free RNA and incomplete RNP complex carrying one L7Ae molecule were eliminated (Fig. 4A, lane 5) in the recovered supernatant containing the non-captured molecules. In contrast, the control experiment (performed by employing a resin on which neither the protein nor the RNA was immobilized), showed no detectable effect on the band patterns (Fig. 4A and 4B, lane 4). Interestingly, the band intensity of the fully loaded triangular RNP was much stronger than that of the input sample, indicating that the treatment with the L7Ae-immobilized resin enhanced the formation of the triangular RNP. This might be due to an increase in the apparent concentration of L7Ae in the system that shifted the equilibrium towards formation of the complex. The free L7Ae remained in the sample after the initial treatment (Fig. 4B, lane 5).

Next, the recovered supernatant was applied onto the fBCD35g-immobilized resin and incubated at room temperature for 5 min. After the treatment, most of the free L7Ae was eliminated (Fig. 4B, lane 6). The triangular RNP was highly enriched although a moderate amount of the incomplete RNP carrying two molecules of L7Ae remained in the sample. The estimated purity and yield were ~80% and ~70%, respectively, and they were sufficient to quantify the fully loaded triangular RNP.

**Fig 5:**
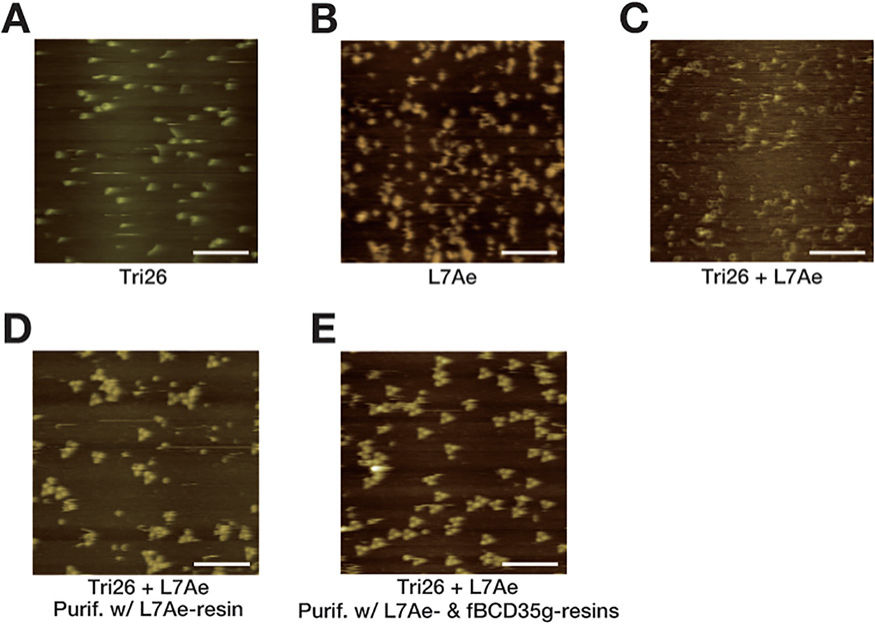
AFM analysis of the triangular RNP purification. AFM was used to visualize Tri26L/S RNA (A), L7Ae protein (B), the crude RNP sample composed of 25 nM Tri26L/S RNA and 75 nM L7Ae (C), the RNP sample after the treatment with L7Ae-immobilized resin (D), and the RNP sample after the treatment with the L7Ae- and fBCD35g-immobilized resins (E). The white bars indicate 100 nm.

To confirm the enrichment of the complete triangular RNP, the purified and non-purified samples were analyzed by liquid-phase high-speed AFM (Fig. 5). After the initial treatment with L7Ae-immobilized resin, the amount of the incomplete RNP and free RNA were dramatically reduced (Fig. 5D). Furthermore, most of the remaining L7Ae was eliminated by the second treatment with fBCD35g-immobilized resin (Fig. 5D). The results are consistent with the data by EMSA (Fig. 4).

### Performance of the purified triangular RNP

In a previous study, the binding affinity of the triangular RNP with multiple antibodies bound was superior to that of the free antibody due to the avidity effect [6]. A similar avidity effect was observed when an affinity peptide against streptavidin, Strep-Tag II (ST2) [7], was bound to the triangular RNP by an L7Ae-ST2 fusion protein (S2 Fig.). When the fully loaded ST2 triangular RNP was constituted with a high concentration of L7Ae-ST2 fusion protein (2 μM) and applied onto a Strep-Tactin (a streptavidin derivative with higher affinity to ST2)-coated plate [15], 1.4% of the input RNA was recovered as a bound fraction (S2 Fig., panel B). The control RNP with only one or two molecules of ST2 (Tri26-m2 and Tri26-m1, respectively) bound showed a lower binding affinity (0.3% and 0.8%, respectively), while the free RNAs showed no binding.

**Fig 6:**
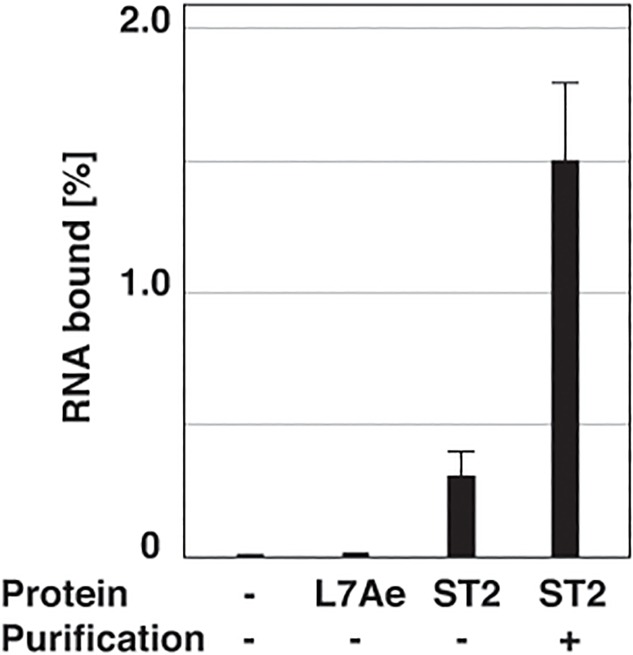
Improved binding profile of the purified sample of the triangular RNP displaying the affinity peptide. The triangular RNP was constituted with 1 nM of [^32^P]-labelled Tri26L/S RNA and 100 nM of L7Ae (2nd lane) or L7Ae-ST2 fusion protein (3rd and 4th lanes). Under these conditions, the RNP sample was a mixture of the complete triangular RNP, incomplete RNP complexes, and free components. As a control, the RNA sample without L7Ae protein was also prepared (1st lane). The triangular RNP with L7Ae-ST2 fusion protein was purified by the sequential affinity elimination (4th lane). The RNP samples were transferred into Strep-Tactin coated plates, and percentage of the bound RNP was determined based on radioactivity of the labeled RNA. The data shown is the average value for the three independent experiments. “ST2” means L7Ae-ST2 fusion protein.

When the triangular RNP was prepared with a lesser amount of the protein (100 nM), the resulting mixture also consisted of the fully loaded triangular RNP, incomplete RNP complexes, and the starting materials (S3 Fig.). In this case, only 0.3% of the input RNA was bound to the plate (Fig. 6), while the free RNA as well as the triangular RNP with L7Ae showed no binding to the plate. After the purification of the ST2-displaying triangular RNP, 1.5% of the input RNA was recovered as a bound fraction, indicating a five-fold increase in binding compared to the mixture. This result demonstrates that enrichment of the complete triangular RNP and removal of the free protein dramatically improves the performance of the triangular RNP. The observed avidity effect was lower than the theoretically expected value. This is perhaps due to the limited concentration of the immobilized ST2 on the plate surface. A better binding effect would likely be observed under optimal conditions for the purification.

## Conclusion

In this study, we developed a new purification procedure for a fully loaded triangular RNP with three proteins. The procedure enables selective purification of the fully loaded triangular RNP from samples containing incomplete RNP complexes that cannot be eliminated by regular affinity purification procedures as confirmed by EMSA (Fig. 4) and AFM (Fig. 5). Consequently, the purification highly improved the performance of the RNP (Fig. 6). This method should be applicable for the purification of other complexes consisting of multiple materials that are constituted *in vitro*. It might also be usable for purifying samples containing unwanted molecules from a mixture such as cell lysates after partial purification by canonical procedures.

## Supporting Information

S1 Fig Sensorgrams of SPR analysis of the original and modified box C/D motifs. Indicated concentrations of BCD35 RNA (A), BCD35a RNA (B), BCD35g RNA (C), BCD35 2’-fPy-RNA (D), BCD35a 2’-fPy-RNA (E), and BCD35g 2’-fPy-RNA (F) were injected onto the L7Ae-immobilized CM5 sensor chip (GE healthcare) at 120 sec. The data was obtained by subtracting the signals for a flow cell without protein immobilization. The analysis was performed by BIAcore 3000 (GE healthcare), and the kinetic parameters were calculated using BIAevaluation software (GE healthcare).
(EPS)

S2 Fig Comparison of binding profiles of the RNPs displaying one, two, or three molecules of the affinity peptide. (A) EMSA analysis of the RNP formation. (B) Binding profile of the ST2-displaying RNPs. Tri26-m1 and Tri26-m2 carry one and two inactively mutated box C/D motifs, respectively; that is, two and one active box C/D motifs are present. All three box C/D motifs of Tri26-m3 are mutated, and L7Ae protein cannot bind to the RNA. The RNPs were constituted with 1 nM of [^32^P]-labelled RNA and 2 μM of L7Ae or L7Ae-ST2 fusion protein, and the binding assay was performed as described in Fig. 6. The data shown are the average value of the three independent experiments. “ST2” means L7Ae-ST2 fusion protein.
(EPS)

S3 Fig Analysis of the RNA and ST2-displaying RNP complexes by EMSA. The ST2-displaying RNP complexes (2nd and 3rd lanes) were prepared with 1 nM of [^32^P]-labelled Tri26L/S RNA (1st and 4th lanes) and the indicated concentrations of L7Ae-ST2 fusion protein. Under the conditions, the resulting mixture consisted of the fully loaded triangular RNP, incomplete RNP complexes, and the starting materials. After the purification by sequential affinity elimination method (5th lane), the fully loaded triangular RNP was highly enriched. The [^32^P]-labelled RNA bands were detected by autoradiography using a phosphoimager screen.
(EPS)
